# The Potential of Bile Acids as Biomarkers for Metabolic Disorders

**DOI:** 10.3390/ijms241512123

**Published:** 2023-07-28

**Authors:** Chang Yin, Ruqing Zhong, Weidong Zhang, Lei Liu, Liang Chen, Hongfu Zhang

**Affiliations:** State Key Laboratory of Animal Nutrition and Feeding, Institute of Animal Science, Chinese Academy of Agricultural Sciences, Beijing 100193, China; yinchang0130@163.com (C.Y.); zhongruqing@caas.cn (R.Z.);

**Keywords:** bile acid metabolism, gut microbiota, enterohepatic circulation, dysbiosis, metabolic disorders, diagnostic biomarker

## Abstract

Bile acids (BAs) are well known to facilitate the absorption of dietary fat and fat-soluble molecules. These unique steroids also function by binding to the ubiquitous cell membranes and nuclear receptors. As chemical signals in gut–liver axis, the presence of metabolic disorders such as nonalcoholic fatty liver disease (NAFLD), type 2 diabetes mellitus (T2DM), and even tumors have been reported to be closely related to abnormal levels of BAs in the blood and fecal metabolites of patients. Thus, the gut microbiota interacting with BAs and altering BA metabolism are critical in the pathogenesis of numerous chronic diseases. This review intends to summarize the mechanistic links between metabolic disorders and BAs in gut–liver axis, and such stage-specific BA perturbation patterns may provide clues for developing new auxiliary diagnostic means.

## 1. Introduction

There have been investigations of bile acids (BAs) since almost three millennia ago, dating back to the widespread use of animal bile in traditional Chinese medicine [1]. Actually, the chemistry and physiology of BAs as a biliary constituent became more detailed by the early 19th century [2]. To begin with, BAs have been widely recognized for their role in promoting the assimilation of dietary fat and fat-soluble substances. Additionally, the presence of micelles within the gall bladder plays a crucial role in dissolving cholesterol in bile. Furthermore, bile salts stimulate the flow of bile from hepatocytes into the bile canaliculi, eventually reaching the gall bladder. Lastly, the conversion of cholesterol to BAs within the liver, followed by the elimination of BAs through fecal excretion, constitutes the primary pathway for cholesterol excretion, which holds paramount importance in maintaining whole-body sterol homeostasis. Since the early 1960s, it has been known that microbes transform human primary BAs to secondary BAs in four different ways via deconjugation of glycine or taurine, as well as dehydroxylation, dehydrogenation, and epimerization of the cholesterol core [3]. And the alterations in the chemistry of these secondary BAs, which were gradually discovered, have been linked to several diseases, such as cirrhosis [4], inflammatory bowel disease [5], and even cancer [6]. Meanwhile, BAs in relation to human disease pathophysiology and treatment have become increasingly indispensable as new BA-based therapies are developed, and through the use of animal models we have enhanced our understanding of BA metabolism and its role in human disease [7]. From then on, there has been much research done regarding the structure, physicochemical properties, metabolism, and physiological function of these essential emulsifiers and signaling molecules. More interestingly, earlier studies have shown that obesity and insulin resistance can be induced by transferring gut microbiota in experimental animals, indicating that alterations of gut microbiota composition and metabolites in humans might be associated with metabolic syndrome [8,9,10]. Indeed, there is a growing awareness that BAs may play more important roles than just as lipid solubilizers or simple regulators of whole-body sterol and BA homeostasis [11]. Although an association between dysbiosis and metabolic diseases is difficult to distinguish, several lines of evidence demonstrate that microbial BA metabolism is crucial for metabolic homeostasis [12,13,14]. Hence, it is unsurprising that several signaling pathways activated by BA have garnered attention as clinical therapeutic targets for metabolic disorders. Besides, BAs themselves hold promise as indicators of health in gut–liver axis [15]. In other words, the dysregulation of BA occurs, which can jointly produce a disease phenotype [16]. Deciphering the pathways of gut microbiota interacting with BAs and altering BA metabolism represents a major public health challenge in the development of new preventive or diagnostic strategies. Here, we review the most significant studies involving host and microbiota-derived BAs in metabolic disorders.

## 2. An Overview of BA Metabolism and Physiology

**Figure 1:**
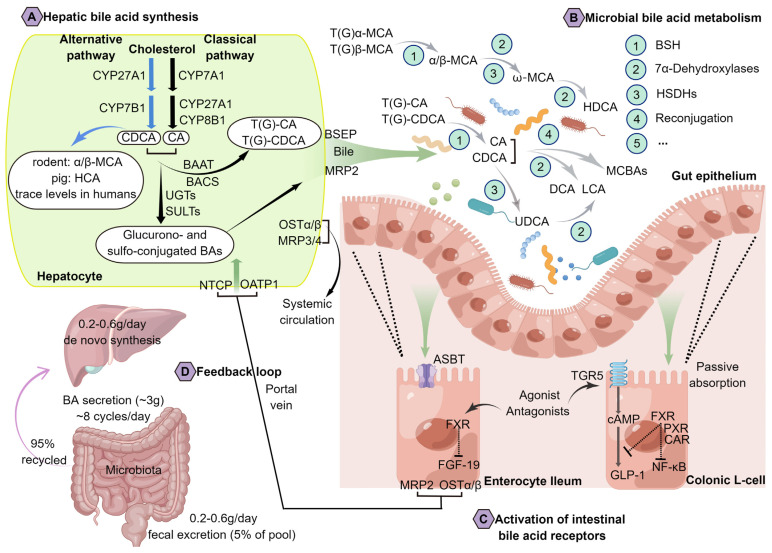
Bile acid metabolism and its enterohepatic circulation.In humans, cholic acid (CA) and chenodeoxycholic acid (CDCA) are the most abundant primary bile acids, while deoxycholic acid (DCA) and lithocholic acid (LCA) are the most abundant secondary bile acids. Hepatocytes synthesize bile acids (via cytochrome P450) by oxidizing cholesterol through two pathways: CYP7A1 initiates the classical pathway and CYP8B1 and CYP27A1 proceed enzymatically to produce the CA and CDCA (mainly in CA), and through CYP27A1 and CYP7B1, an alternative pathway yields CDCA [12,18]. In the rodent liver, the majority of CDCA is transformed to α-muricholic acid (α-MCA) and β-MCA; in the pig, CDCA is primarily transformed to hyocholic acid (HCA) by CYP4A21, whereas in humans it remains as CDCA [23,24,25]. Hepatocytes conjugate most bile acids to glycine (glyco-, G) or taurine (tauro-, T) before secreting them into bile via bile salt export pump (BSEP) [7]. Meanwhile, sulfated (sulfo-) or glucuronidated (glucurono-) bile acids produced by sulfotransferases (SULTs) and UDP-glucuronosyltransferases (UGTs) amidated with a taurine or a glycine are then taken up via the multidrug resistance-associated protein 2 (MRP2) [16]. In the intestine, glyco-conjugated and tauro-conjugated CA and CDCA are deconjugated via bile salt hydrolases (BSH) and 7α-dehydroxylases to produce secondary bile acids (DCA and LCA) [22]. α-MCA and β-MCA are largely conjugated with taurine, among them, β-MCA is C-6 epimerized to form ω-MCA and then ω-MCA is 7α-dehydroxylated to from HDCA [12]. The enzyme hydroxysteroid dehydrogenase (HSDH) transforms CDCA into UDCA. Indeed, recent research has revealed that bile acids can be reconjugated into conjugated forms (microbially conjugated bile acids, MCBAs) by gut microbiota [30]. MRP2 is the main excretion pathway for glucurono- and sulpho-conjugated BAs. At the terminal ileum, most of the unconjugated BAs are reabsorbed by apical sodium-dependent BA transporter (ASBT) into the enterocytes, and through the basolateral bile acid transporters, organic solute transporters (OST-α/β) and MRP2/3, secreted into the portal circulation. By transporting bile acids into hepatocytes through sodium-dependent taurocholate co-transporting polypeptide (NTCP) and organic anion-transporting polypeptide 1 (OATP1), hepatic MRP3/4 and OST-α/β also provide routes of alternative excretion for bile acids into the systemic circulation [16,19].

The conversion of cholesterol into BAs contributes to cholesterol homeostasis, approximately two-fifths of the body’s excretion of cholesterol occurring through BA formation. Previous research has shown that a decrease in BA concentrations can reduce cholesterol solubility, promoting it to form microcrystals, which cause cholelithiasis with diminished serum BA concentrations [17]. Moreover, a fraction of cholesterol is employed for excretion into the bile, while another fraction may undergo esterification via acyl CoA: cholesterol acyltransferase, leading to its integration into the nascent very-low-density lipoprotein (VLDL) particle and subsequent release into the plasma compartment. Primary BAs (such as CA and CDCA) can be synthesized de novo from cholesterol via two pathways (the classical and alternative pathways, as shown in Figure 1A) in hepatocytes through cytochrome P450 (CYP) enzymes, which include at least seventeen different enzymes, such as cholesterol 7 alpha-hydroxylase (CYP7A1), oxysterol 7 alpha-hydroxylase (CYP7B1), sterol 12 alpha-hydroxylase (CYP8B1), sterol 27-hydroxylase (CYP27A1), and so on. Among them, the enzyme CYP7A1 is responsible for limiting the rate. One or more transporters are also involved, as well as multiple compartments within the cell, including the cytosol, endoplasmic reticulum, mitochondria, and peroxisomes [18]. Since the biosynthetic intermediates in these pathways are often substrates for more than one enzyme and little is known regarding the intracellular trafficking of these compounds in hepatocytes, the mechanisms responsible for the metabolic channeling of cholesterol toward such primary BAs remain unclear [7]. In general, BAs consist of four steroid rings arranged in a hydrocarbon lattice with hydrophobic convex faces and hydrophilic concave faces, and an acidic side chain of five carbons is then amidated with taurine or glycine [19]. As a result of this amphipathic structure, BAs have the detergent properties that enable them to form micelles. Therefore, it is conventionally postulated that BAs exert a significant role in facilitating the uptake of nutrients by facilitating the amalgamation of emulsified lipids with polar phospholipid molecules via a non-receptor-mediated mechanism, thereby promoting the emulsification and absorption of lipids and fat-soluble vitamins within the small intestine [20].

BAs are amidated (“conjugated”) with glycine or taurine before they are excreted into the bile and passed to the duodenum, and there seem to be considerable differences in binding characteristics among species. For instance, BAs are mostly conjugated with glycine in humans, but in mice and rats are almost exclusively conjugated with taurine [12]. In response to food intake, the gallbladder releases BAs directly into the duodenum through cholecystokinin (CCK) stimulation. Despite the lack of complete understanding of the chemistry of BA reabsorption, it is generally understood that approximately 95% of biliary secreted BAs (predominantly as conjugated BAs) are reabsorbed from the terminal ileum by the apical sodium-dependent bile acid transporter (ASBT), and recirculated via the portal vein to the liver, where they are secreted again [18]. Enterohepatic circulation refers to this process and occurs in normal adults approximately 8 times daily, the BA pool of about 3 g is recycled in gut–liver axis, and it takes only 0.2–0.6 g of de novo synthesized BAs each day to sustain the BA pool stable [16]. And the extensive reviews had been conducted elsewhere on BA synthesis, transport, metabolism, and regulation [12,16,18]. Significantly, the metabolism of BAs is profoundly impacted by the gut microbiota, as BAs undergo partial conversion into bioactive microbial metabolites within the intestinal lumen. More specifically, primary BAs that evade reabsorption in the distal ileum are subsequently metabolized into secondary BAs, such as DCA, LCA, UDCA, and others [21]. Although the bacteria-derived secondary BAs constitute only a small percentage of normal BA production, it leads to increased diversity and in general a more hydrophobic BA pool, which facilitates fecal elimination of BAs, less than 5%. In addition, hydroxyl groups at positions 3, 6 (C6, observed in MCA), 7, and 12 on the steroid backbone affect both their solubility and hydrophobicity (LCA, DCA, CDCA, CA, UDCA, and MCA are the order of hydrophobicity, for example) [22]. Thus, several BA-responsive receptors are activated with different specificities due to these small structural differences (unconjugated) [19]. Based on previous studies, β-muricholic acid (β-MCA), a major BA in rodents, acts as an antagonist of farnesoid x receptor (FXR) [23]; hyocholic acid species (HCAs) are major BAs in pigs which can specifically activate the cell membrane receptor G protein-coupled bile acid receptor 1 (TGR5, also known as GPBAR1) [24]; CDCA, a ubiquitous component found in the BA pools of mammals, which is a strong agonist of FXR [25]. These molecules serve as crucial signal molecules that activate cell signaling pathways, thereby regulating glucose and lipid metabolism. Additionally, they play a protective role in preventing intestinal barrier dysfunction and endoplasmic reticulum damage [26,27,28,29]. Over the past two decades, the understanding of BA signaling has been greatly improved, and most of the basic research and therapeutic exploration has been focused on FXR and TGR5, the two most well-characterized receptors for BAs.

## 3. Bacterial Metabolism of BAs Has Been Recognized as an Important Actor within the Metabolic Homeostasis of the Host

A variety of symbiotic bacteria and other microorganisms colonize the human gastrointestinal tract, collectively known as the gut microbiota [31]. The co-production of a diverse array of metabolites, serving as energy substrates and/or signaling factors, is facilitated by the interaction between the host genome and the microbiome. There has been an increasing interest in BAs, which play an important role in digestive processes or act as the critical mediators of intercellular communication in gut–liver axis [32]. Amongst others, choline is essential for the transportation of lipids, the methylation of proteins, and the synthesis of neurotransmitters such as glycine, glutamate, and gamma aminobutyric acid (abbreviated as GABA) [33,34]; short-chain fatty acids (SCFAs) including acetate, propionate, and butyrate, are now a well-recognized connection between the gut microbiota and host cells [34]. Interaction between the host and this dynamic set of small-molecule metabolites regulates the immune system and metabolic phenotypes, also confers the observed disease risk. Prior research indicates that intestinal BAs are transformed into the unconjugated forms by gut microbiota, which is crucial to gastrointestinal homeostasis (as shown in Figure 1B,C) [16]. Although we do not fully understand the specific chemistry behind how BAs are modified and reabsorbed, it is widely accepted that certain bacterial genera play key roles. These genera, including *Bacteroides*, *Lactobacillus*, *Clostridium*, *Listeria*, and *Bifidobacterium* are involved in the deconjugation reaction of tauro- and glyco-conjugated BAs through the enzyme bile salt hydrolase (BSH). Besides, several genera such as *Bacteroides*, *Clostridium*, *Escherichia*, *Eubacterium*, *Eggerthella*, *Eubacterium*, *Peptostreptococcus*, and *Ruminococcus* are capable of oxidizing and epimerizing the hydroxyl groups at C3, C7, and C12 [16]. Among them, the 7α-dehydroxylation process is carried out by *Clostridium* and *Eubacterium*; the esterification of BAs occurs through *Bacteroides*, *Eubacterium*, and *Lactobacillus*; and the desulfation occurs through *Clostridium*, *Fusobacterium*, *Peptococcus*, and *Pseudomonas* [18,35]. Aside from the previously known transformations, recent research has indicated that microbiota-mediated BAs were re-conjugated with leucine, tyrosine, or phenylalanine at the position of C24 acyl by *Enterocloster bolteae* (formerly *Clostridium bolteae*), was similar to the usual conjugation mechanism of the host [30,36]. However, additional mechanistic investigations are required to enhance the comprehension of these microbial metabolites.

## 4. Major Factors Associated with Alterations in BA Metabolism

### 4.1. Antibiotics

There is sufficient evidence that the gut microbiota affects not only the BA profile and pool size but also the expression of genes controlled by the BA-activated receptor FXR. For example, in germ-free rats, there was a decrease in serum and intestinal BA concentrations, and a slight reduction was observed in rats subjected to antibiotic treatment that only partially diminished the microbiome [37]. Specifically, the uncoupling and conversion to the secondary BAs pathway was blocked in the gut, and changes in the hepatic expression of genes regulated by the BA-responsive nuclear receptor FXR [37]. Another previous study pointed out that short-term treatment of mice with non-absorbable antibiotics, such as vancomycin and polymyxin B, resulted in a reduction in abundances of BA modification-associated gut microbiota, the contents of hepatic secondary BAs and circulating triglyceride were also decreased, this suggests the secondary BAs as regulators are also involved in the maintenance of metabolic homeostasis [38].

### 4.2. Diet

Distinct dietary patterns can affect the BA pool directly or indirectly. For example, a high-fat diet (HFD) stimulates hepatic BA synthesis and secretion, and induces alterations in the intestinal flora [39]. This is due to the potent antimicrobial properties typically exhibited by BAs. Numerous studies therefore have investigated the sensitivity or resistance of probiotics to BAs to facilitate industrial applications of probiotics, as it is considered one of the main toxic challenges that probiotics must be able to survive bile exposure [39]. Furthermore, it has been found that oral probiotics modified the gut microbiota profile of mice, resulting in increased BA excretion in feces and increased BA production in the liver through decreased BA reabsorption from the terminal ileum and inhibition of the enterohepatic FXR-fibroblast growth factor 15 (FGF15) axis [40], which indicates that microbial dysbiosis and abnormal BA metabolism might be reversed with probiotics. Similar findings were observed in a pig model, dietary fiber with different types had diverse effects on microbial metabolites BAs and their related receptors expressions by altering the composition of gut microbiota [41]. It is generally believed that dietary fibers can exert beneficial anti-inflammatory effects via microbial metabolites including SCFA and so on [42]. However, it has recently been shown that dietary intake of inulin fiber affects the composition of the microbiota and microbiota-derived metabolites, particularly in relation to BAs, which play a crucial role in promoting type 2 inflammation [43].

### 4.3. Disease

#### 4.3.1. Chronic Liver Diseases Such as NAFLD and NASH

Prior studies conducted on rats have shown that the nonalcoholic fatty liver disease (NAFLD) induced by a HFD resulted in an elevation in the hepatic TCA composition, while the levels of THDCA and UDCA were found to be reduced [44]. An additional recent study has found significantly elevated values of serum GCA to TCA ratio, GDCA to TDCA ratio, as well as GCDCA to TCDCA ratio in patients with early chronic liver disease, such as NAFLD and nonalcoholic steatohepatitis (NASH) [45]. Furthermore, another study revealed a notable elevation of various conjugated 12-OH BAs, particularly those derived from the microbiota such as TDCA and GDCA, within the serum of individuals diagnosed with NASH [46]. Further studies demonstrated that 12-OH BAs (administration of GDCA and TDCA) significantly increased hepatic stellate cell proliferation and protein expression of fibrosis-related markers, blockade of BA binding to TGR5 or inhibition of extracellular signal-regulated kinase (ERK1/2) and p38 mitogen-activated protein kinase (MAPK) signaling, both significantly attenuated the BA-induced fibrogenesis, using a liver fibrosis mouse model [46,47].

#### 4.3.2. T2DM

Previous research has demonstrated that alterations in gut microbiota associated with type 2 diabetes mellitus (T2DM) may potentially lead to decreased levels of HCA species, as the metabolism of HCA species is closely associated with gut microbiota in humans [48]. HCA and its derivatives are the major BA species in the BA pool of pigs (more than 60%), and human blood and feces also contain small amounts (~3%), one species is known for its exceptional resistance to the development of spontaneous diabetic symptoms [24]. There is a relationship between T2DM and lower levels of HCAs in both serum and feces, demonstrating the feasibility of using HCA profiles to assess the future risk of developing metabolic abnormalities [49]. Besides, serum HCA levels were increased in the patients after gastric bypass surgery, and it was subsequently demonstrated that HCA levels can predict the remission of diabetes two years after surgery [49].

#### 4.3.3. Gastric and Hepatocellular Cancer

Accumulating evidence reveals that the microbially transformed BAs are strongly associated with gastric (GC) and hepatocellular (HCC) cancer risk, development, and progression. For example, bile reflux gastritis induced by LPS-producing bacteria (*Prevotella melaninogenica*) is associated with the development of GC, and subsequently also identified that the secondary BA, TDCA, is significantly and positively correlated with the LPS-producing bacteria in the gastric juice of both bile reflux gastritis and gastric cancer patients [50]. And increased amounts of CDCA are also detected in advanced liver disease, including HCC, due to the upregulation of the alternative BA synthesizing pathway [51]. An observational study of changes in serum BAs in different chronic liver disease stages revealed that patients with early-stage HCC and cirrhosis exhibited a higher level of circulating TBA, mainly increased conjugated primary BAs including conjugated CA and CDCA, and increased TUDCA in comparison with the same stage of cirrhosis patients without HCC [52]. Similarly, another metabolomics study found that patients with HCC had the increased CDCA levels in both serum and tumor tissue, which was considered as a potential biomarker [53], despite the function of elevated CDCA in HCC remains controversial.

#### 4.3.4. Enteric Diseases

It appears that colorectal cancer (CRC) patients with a Western-style diet (a diet high in saturated fats and refined carbohydrates) have higher levels of secondary BAs in both serum and feces, mainly in DCA and LCA [54]. Increased levels of such secondary BAs exert detrimental effects on the architecture and function of the colonic epithelium through multiple mechanisms as described previously [54]. However, several other studies of enteric diseases found a substantial drop in the abundance of the phylum *Firmicutes*, and a decline in the ratio of *Faecalibacterium prausntizii* to *Escherichia coli*, as well as markedly higher levels of fecal conjugated BAs and lower levels of secondary BAs, including *3-oxo*-LCA and *isoallo*-LCA in active IBD patients compared with healthy individuals [55,56]. Interestingly, research regarding new BA conjugations revealed that phe-CA, tyr-CA, and leu-CA were enriched in patients with IBD [36]. In addition, the elevated 3α-OH-sulfated secondary BAs were also detected in feces of active IBD patients, which are more hydrophilic to reduce cytotoxicity and increase excretion in urine and feces [57].

#### 4.3.5. Cholestatic Liver Diseases

In addition, primary sclerosing cholangitis (PSC) and primary biliary cirrhosis (PBC) are cholestatic liver diseases ultimately resulting in cirrhosis and liver failure [58]. According to reports, there was an increase in primary BAs such as CA, TCA, CDCA, and TCDCA, while the level of secondary BAs decreased in the serum and feces of patients with PBC [59], and there seems to have been a blockage in the transformation of primary BAs into secondary BAs in the gut. PSC is highly comorbid with IBD (approximately 80% of all PSC patients), and it is associated with elevated risks of cholangiocarcinoma and CRC [60]. BA profiling revealed a statistically increased level of serum TBA, mainly in CA, CDCA, and their conjugates as well as UDCA while the secondary BAs including LCA, TLCA, DCA, and GDCA were decreased except for the increased level of TDCA in PSC patients [61,62]. The gut microbiota–FXR–FGF15 axis plays a crucial role in PSC pathophysiology via modulation of BA synthesis, as a result of the loss of microbiota-mediated negative feedback control of BA synthesis, the concentrations of BA in the liver and circulatory system increased (particularly in CA, CDCA and their conjugates, UDCA, TDCA levels), causing disruption of bile duct barrier function, which leads to fatal liver injury [63]. Besides, the presence of an excessive accumulation of BAs in the maternal and fetal serum, referred to as intrahepatic cholestasis of pregnancy (ICP), contributes to the heightened likelihood of preterm delivery, fetal distress, and spontaneous abortions during gestation. One large retrospective cohort study in China, encompassing 68,245 singleton pregnancies, and analysis revealed that maternal circulating TBA concentration of 4.08 μg/mL or above was positively associated with an increased risk of low birth weight and intrauterine growth restriction, the risk appeared to be higher when coexisting with hypertensive disorders during pregnancy [64]. It seems that clinicians should monitor BA concentration during the follow-up for pregnancies with potential intrauterine growth restriction. Nevertheless, the generalizability of these findings remains uncertain due to the dearth of research conducted across different regions and countries.

The aforementioned findings regarding the variations in BA profiles among different patients have been consolidated and presented in Table 1.

## 5. BAs Shape the Gut Microbiota

BAs especially unconjugated forms are potent antibacterial compounds and play an important role in shaping gut microbial ecology, and also affect host metabolism indirectly [72]. The detergent activity of BAs can directly and rapidly affect bacterial global metabolism through disrupting cell membranes, damaging DNA, altering the conformation of proteins, and chelating iron and calcium. Furthermore, it has been observed that gram-positive bacteria exhibit a higher sensitivity to these effects [72]. Another research found the secretion of tryptophan-derived antibiotics (turbomycin A and 1-acetyl-β-carboline) by BA 7α-dehydroxylating bacteria, *C. scindens* and *C. sordellii*, both antibiotics function by inhibiting the growth of *C. difficile* and other gut bacteria [73,74]. And the secondary BAs, DCA and LCA, but not CA, enhanced the inhibitory activity of these antibiotics [75]. This implies that the bacteria colonizing the intestinal tract must have BA tolerance properties and mechanisms for repairing BA damage. Hence, certain strains of probiotic bacteria, namely *Lactobacillus* and *Bifidobacterium*, along with 7α-dehydroxylating bacteria such as *C. scindens*, demonstrate resistance to BAs through the activation of glycolysis [72]. The microbial modification of BAs plays an equally important role in maintaining a healthy gut microbiome and preventing diseases. Thus, well-balanced interactions between gut microbiota and host play a vital role in safeguarding against pathogen colonization, and our comprehension of how BA modification contributes to this protective effect is just emerging.

## 6. BAs Are Closely Associated with Metabolic Disorders

### 6.1. Dysregulation of BA Synthesis Contributes to Metabolic Disorders

**Figure 2:**
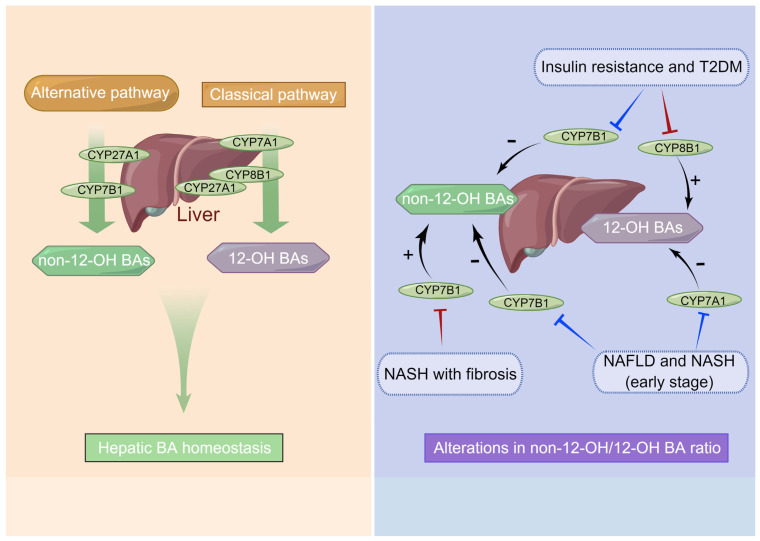
The dysregulation of bile acid synthesis in metabolic disorders. Bile acid (BAs) synthesis is altered in patients with nonalcoholic fatty liver disease (NAFLD) and type 2 diabetes mellitus (T2DM) and is associated with hepatic steatosis and inflammation, which contribute to liver injury. Specifically, insulin resistance and T2DM are associated with upregulated CYP8B1 and downregulated CYP7B1. While NAFLD and nonalcoholic steatohepatitis (NASH) without fibrosis are accompanied by downregulated CYP7A1 and CYP7B1 expressions and a consequent decline in biotransformation of cholesterol into bile acids. However, the alternative BA synthase pathway is upregulated in NASH with fibrosis. Thus, the balance between CYP8B1 and CYP7B1 activity may determine glucose and lipid metabolism homeostasis via analyzing bile acid ratios. Note: the non-12-OH bile acids, mainly including chenodeoxycholic acid (CDCA), α/β-muricholic acid (MCA), hyocholic acid (HCA), ursodeoxycholic acid (UDCA), lithocholic acid (LCA), along with their conjugates derived from the alternative pathway, and the 12-OH bile acids, including cholic acid (CA), deoxycholic acid (DCA), and their conjugates derived from the classical pathway (**left**); red arrows indicate upregulated and blue arrows indicate downregulated (**right**).

As dietary habits and lifestyles change, the incidence of fatty liver disease is increasing among the worldwide population, such chronic liver disease has become the second largest liver disease after viral hepatitis, and it also increases the risk of developing insulin resistance, T2DM, and cardiovascular disease [76]. Notably, it is estimated that approximately 25% of adults suffer from NAFLD, and about 2% to 6% suffer from NASH [77]. Statistics from developed countries show that nearly a quarter of patients with NASH will develop hepatic fibrosis and cirrhosis within the next twenty years, which can contribute to fatal liver diseases [77]. The related study previously indicated that individuals with hyperlipidemia who exhibited increased levels of circulating TBA demonstrated higher body mass indexes and serum triglyceride levels [78]. Human metabolic disorders such as insulin resistance and even T2DM have been linked to elevated 12-OH BAs, which are synthesized from the classical pathway and regulated by CYP8B1, such as CA, DCA, and their conjugates (see Figure 2) [66]. While CYP8B1 depletion left hosts with a higher ratio of non-12-OH BAs mainly includes CDCA and its derivatives synthesized from the alternative pathway, which was beneficial for their metabolic health [79]. Indeed, the expression of hepatic CYP7B1 was found to be diminished in animal models of diabetes and NAFLD, implicating the alternative pathways may contribute to human metabolic homeostasis [67,68]. It was also reported that obese T2DM patients displayed a higher ratio of serum CDCA before Roux-en-Y gastric bypass surgery which was associated with a shorter duration of T2DM, and the CDCA levels at baseline were also associated with better diabetes remission rates post-surgery [80]. Moreover, hepatic injury gradually upgrades the accumulation of free cholesterol, NAFLD and NASH are characterized by an increase in intracellular cholesterol due to downregulated CYP7A1 expression and a consequent decrease in biotransformation of cholesterol into BAs [65]. Still other reports demonstrated that expression of hepatic CYP7B1 was reduced in NAFLD, early NASH, and T2DM, but NASH with fibrosis may lead to an upregulation of the alternative BA pathway caused by activated CYP7B1 expression [81,82]. The alternative BA synthase pathway is upregulated in late-stage chronic liver diseases with fibrosis and cirrhosis, and it has been believed to be a protective (or compensatory) mechanism to alter BA profiles by synthesizing higher concentrations of hydrophilic (non-12-OH) BAs, in which the host excretes them more readily [51]. Thus, the analysis of BA ratios can potentially elucidate the role of CYP8B1 and CYP7B1 activity in maintaining homeostasis of glucose and lipid metabolism.

### 6.2. Gut Microbiota Interacting with BAs and Altering BA Metabolism Are Critical in Metabolic Disorders

**Figure 3:**
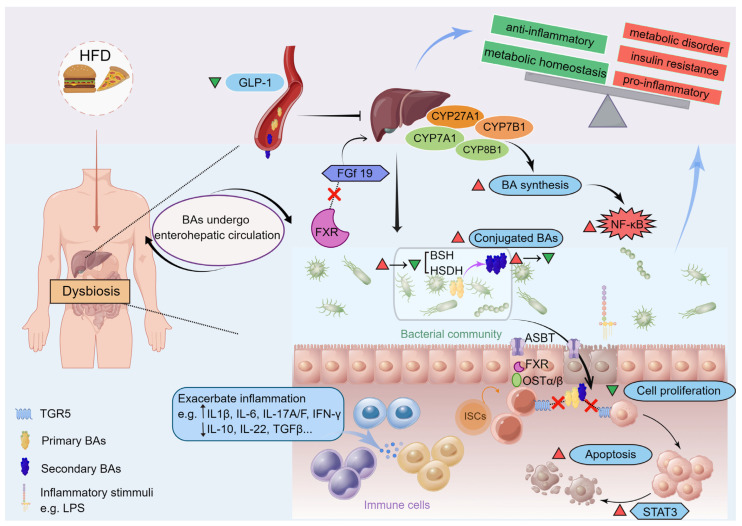
Gut microbiota interacting with bile acids and altering bile acid metabolism are critical in metabolic disorders. Metabolic health is determined by long-term dietary habits via alterations in gut microbiota and bile acid (BA) composition. As implied by the findings described above, a high-fat diet (HFD) induces a higher level of secondary bile acids in the lower intestine due to gut microbiota alteration (exhibit an elevated level of aerobic, proinflammatory bacteria, as well as bile acid modification-associated bacteria), which increases intestinal reabsorption and liver bile acid return. Subsequently, several major bile acid transporters (including bile salt export pump, BSEP) are inhibited by increased liver inflammation and downregulation of liver FXR and its target genes via activation of nuclear factor-κB (NF-κB). This results in a reduced transport of bile acids from the enterocyte back into the portal and therefore causes bile acid accumulation in hepatocytes and intestinal mucosa. On the other hand, evidence suggests that HFD-induced elevated bile acids in the gut might be temporary, and the unconjugated BAs might be progressively depleted from the gut under conditions of chronic inflammation, probably due to the depletion of BSH-rich bacteria in the gut. Secondary bile acids are shown to be involved in the sustenance of a viable epithelial layer via inducing apoptosis and cell turnover [97]. However, high levels of secondary bile acids following HFD intervention and then gradually decreasing levels following inflammation might disturb epithelial barrier function via activation of STAT3 in the gut, and lost FXR function as a consequence, resulting in the loss of negative feedback regulation (FXR-FGf19) of bile acid synthesis [16,55]. Besides, this process may be accompanied by a reduction of glucagon-like peptide-1 (GLP-1) secretion [24,27]. Thus, it seems that the disturbed gut microbiota-dependent bile acid metabolism, subsequently detected through qualitative or quantitative alterations of the BA profiles, is a prevalent risk factor for metabolic disorders. Note: red triangles indicate upregulation and green triangles indicate downregulation. ASBT, apical sodium-dependent BA transporter; BSH, bile salt hydrolase; FGf19, fibroblast growth factor 19; HSDH, hydroxysteroid dehydrogenase; ISCs, intestinal stem cells; LPS, lipopolysaccharide; OSTα/β, organic solute transporter alpha/beta; STAT3, signal transducer and activator of transcription3.

Meanwhile, the interplay between the gut microbiota and the BA pool is greatly impacted by the host diet, as illustrated in Figure 3. It is reported that consumption of high fat, especially saturated fat promotes the upregulation of hepatic 12-OH BAs such as TCA, resulting in an increased abundance of sulfite-reducing bacteria such as *Bilophila wadsworthia*, causing increased susceptibility to DSS-induced colitis in IL-10^−/−^ mice, and more severe hepatic steatosis, disrupted glucose metabolism and barrier dysfunction after HFD treatment [83,84]. As previously reported, the increased primary BAs including CA and CDCA concentrations induces the decreased VLDL secretion and serum triglyceride content via the FXR-sterol regulatory element binding protein-1c (SREBP1c) pathway [85]. The presence of hepatic lipids, including cholesterol and triglycerides, has therefore been observed to accumulate in a mouse model lacking FXR when fed a normal chow diet. Conversely, in obese individuals or those consuming excessive fat, the absence of FXR has been found to enhance glucose homeostasis, resulting in weight loss. This effect may be attributed to changes in the gut microbiota composition [86]. According to studies in conditional knockout mice, it appears that FXR as one of the main BA receptors may play different roles in different tissues during metabolic disorders. Early research indicated that a feedback loop happens though FXR drives the expression of FGF19 in intestinal epithelial cells, which inhibits de novo synthesis of hepatic BAs, while mice overexpressing FGF19 leads to protection against HFD-induced metabolic injury via increasing metabolic activity and energy expenditure [87].

In metabolic disorders, such alteration in BA metabolism might contribute to chronic low-grade intestinal inflammation, as the conjugated primary BAs exhibit proinflammatory roles on the intestinal epithelium, which may contribute to obesity and atherosclerosis [55]. However, there is debate as to whether secondary BAs have anti-inflammatory effects, which may depend on the context. It is generally accepted that BAs can modulate host metabolism by signaling through their receptor-mediated pathways, and the different basal gut microbiota may explain this phenomenon. Besides, gut microbiota perturbations could lead to pathology, especially in diseases related to metabolism and autoimmunity, and induce impairment reabsorption of BAs in the ileal, which normally occurs via ASBT, resulting in decreased expression of FXR and FGF19 and an imbalance of BAs, notably characterized by an increase in hepatic and colonic primary conjugated BAs [88]. Therefore, patients with inflammatory bowel disease (IBD) often suffer from gut microbiota dysbiosis accompanied by microbial diversity declines and a lower relative abundance of *Firmicutes*, leading to the impaired function in metabolizing primary BAs via a reduction in BSH and HSDH activity [16,86]. In addition, the microbiota-induced changes in BA profiles also could cause diminished hepatic FXR activity, decreased bile salt export pump (BSEP) and OSTα-OSTβ complexes (OSTα/β) expressions, and so on. [89,90]. Hepatocytes and intestinal mucosa are subsequently damaged due to increased intracellular retention of BAs [16]. It was also noted that increased conjugated BAs correlate with several other intestinal disorders, including ulcerative colitis, Crohn’s disease (CD), and irritable bowel syndrome (IBS) [69].

Patients with fatty liver disease, fibrosis, cirrhosis, and even HCC typically exhibit gut microbiota dysbiosis with an elevated level of aerobic, proinflammatory bacteria, BSH-rich *Enterobacter*, *Enterococcus* and *Clostridium bacteria*, which produce secondary BAs in greater quantities [18,91]. Thus, it seems that the disturbed gut microbiota-dependent BA metabolism, and subsequently detected through qualitative or quantitative alterations of the BA profiles, is a prevalent risk factor for nearly all liver diseases [92,93,94]. As implied by the findings described above, a higher level of secondary BAs in the lower intestine was detected with HFD due to gut microbiota alteration, which improved intestinal reabsorption and liver BA return [95]. Subsequently, several major BA transporters, such as BSEP, experience inhibition due to heightened liver inflammation and the downregulation of liver FXR and its target genes, which is facilitated by the activation of nuclear factor-κB (NF-κB) [92]. This results in the diminished transportation of BAs from the enterocyte back into the portal vein via the OSTα/β efflux complex and ileal BA-binding protein (IBABP) and leads to the accumulation of BAs in hepatocytes and intestinal mucosa [96]. In studies of obesity-related CRC, a HFD diet also increased the risk of CRC in humans, this might be due to the increased production of secondary BAs in the colon [71]. There is, however, evidence suggesting that HFD-induced the elevated BAs in the gut might be temporary, and the above-mentioned unconjugated BAs might be progressively depleted from the gut under conditions of chronic inflammation, probably due to the depletion of BSH-rich bacteria such as *Lachospiraceae*, *Clostridiaceae*, *Erysipelotrichaceae*, and *Ruminococcaceae* families in the gut [55,70]. Secondary BAs are shown to be involved in the sustenance of a viable epithelial layer via inducing apoptosis and cell turnover, however, high levels of secondary BAs following HFD intervention and then gradually decreasing levels following inflammation might have two important consequences. First is an increase in apoptosis of epithelial cells with high expression levels of FXR on the apical surface [97]. The second aspect pertains to sustained epithelial cells renewal and improved selective growth of less differentiated cells that lower FXR expression and are resistant to apoptosis [98]. As a result of gut dysbiosis, unconjugated secondary BAs are gradually depleted, and the colon epithelium became less differentiated, highly proliferative, and lost FXR function as a consequence, which may contribute to cancer initiation.

### 6.3. Microbiota-Derived BAs Serve as the Chemical Signals and Biomarkers in Gut–Liver Axis

**Figure 4:**
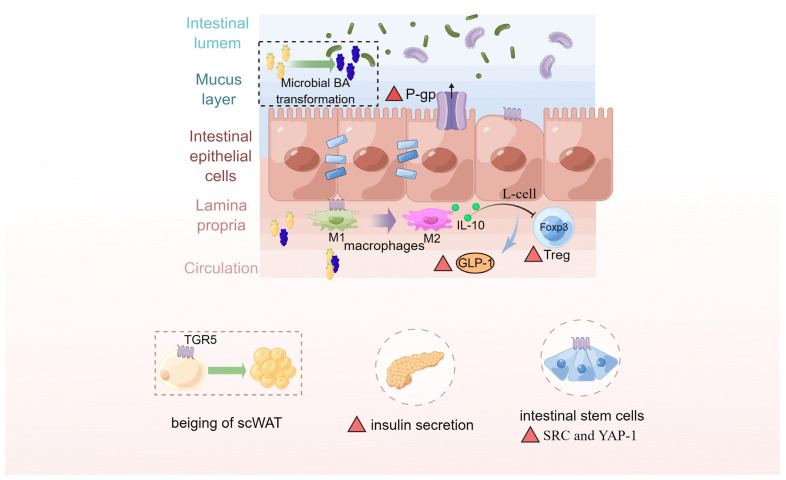
Microbially transformed bile acids exert profound beneficial roles on host pathology via activation of TGR5. G protein-coupled bile acid receptor 1 (TGR5, also known as GPBAR1) is activated mainly by microbiota-derived bile acids (BAs) such as lithocholic acid (LCA) and its derivatives, leads to regulate energy and glucose homeostasis, lymphocyte development, immune responses, inflammation, cell proliferation, and so on. As described above, beige fat remodeling occurs in subcutaneous white adipose tissue (scWAT) that may further improve energy homeostasis throughout the body via activation of BA-TGR5 pathway. Bile acids especially in hyocholic acid species (HCAs) can promote GLP-1 secretion by activating TGR5 and inhibiting farnesoid x receptor (FXR). Activation of TGR5 also links to P-glycoprotein (P-gp) expression, which mediates the efflux of drugs/xenobiotics from the intestinal mucosa into the gut lumen to protect the intestinal epithelium. Bile acids induce activation of tyrosine kinase (SRC) and Yes-associated protein 1 (YAP-1) via TGR5 in intestinal stem cells (ISCs), thereby facilitating the renewal of ISCs and promoting regeneration in response to injury. Mucosa-associated macrophage phenotypes switch from M1 (pro-inflammatory) to M2 (tissue-protective) via activation of TGR5. As a result of these anti-inflammatory functions, BA-dependent TGR5 signaling promotes the development, recruitment, and expansion of Foxp3+ T regulatory (Treg) cells in the gut. Note: red triangles indicate upregulation.

In both human and animal models, there is growing evidence that the dysregulation of BA metabolism is associated with metabolic disorders and has therefore linked the BAs to several chronic inflammatory diseases [54,99,100]. As discussed above, microbially transformed BAs including several non-12-OH BAs also exert profound impact on host pathology due to their pleiotropic roles in metabolism and immune function [101]. Among them, the 7β-epimer of CDCA is important for producing the TUDCA and HCA species, which are well-characterized BAs known to maintain glucose homeostasis via activation of TGR5 [24,102]. In recent years, TGR5 has gained increasing attention, which displays a strong affinity for microbiota-derived BAs [103,104,105]. To the best of our knowledge, TGR5-related pathways are not completely understood, but TGR5 immunohistochemical staining has been observed in twelve percent of human intestinal metaplasias, that is therefore believed bile reflux is a risk factor in the development of gastric carcinogenesis [16,50]. In addition, it has been reported that GLP-1 and insulin are secreted at increased levels in transgenic mice overexpressing TGR5 [20,92]. As a result of BA-TGR5 pathway activation, beige fat remodeling occurs in subcutaneous white adipose tissue, that may further improve energy homeostasis throughout the body [106]. In patients suffering from ulcerative colitis, diminished *P-glycoprotein* expression coupled with the reduction of TGR5-specific BA content with a reduced capability to induce *P-glycoprotein* expression [107]. By injecting CCK into mice, Sorrentino, et al. [108] found that BAs promoted the growth of intestinal organoids (activation of the SRC-YAP pathway), more specifically, TGR5 expression in intestinal stem cells was required for homeostatic intestinal epithelial renewal and fate specification, as well as for regeneration after colitis induction. Furthermore, activation of macrophages’ BA-responsive TGR5 is known to regulate cytokine production in the gastrointestinal tract [109]. BA receptor TGR5 has therefore become an attractive promising target for immune modulation, and several agonists have entered clinical trials, aiming to offer new treatment options for several diseases. Moreover, UDCA exhibits protective effects in the gut, specifically through inhibition of TNFα, IL-1β, and IL-6 release [110,111,112]; TLCA and TUDCA mediate mucosal humoral immune responses to limit the development of IBD via inhibition of apoptosis [113]. There is also evidence that the other microbiota-derived BAs such as *isoallo*-LCA, *iso*-DCA, and *3-oxo*-LCA can modulate the function and differentiation of T cells including both inflammatory T helper 17 (TH17) cells and anti-inflammatory regulatory T (Treg) cells to maintain host immune tolerance and protect against extracellular pathogens [56,114]. (See Figure 4).

On the other hand, researchers have been motivated to assess the relationship between BA profiles or BA species ratios and the development of human metabolic disorders as a result of their physiological and metabolic importance. Over the last few decades, metabolism-related disorders, such as obesity, NAFLD, and T2DM have reached epidemic proportions globally and have become an increasingly important field of clinical and translational research [115]. However, common biomarkers used for early detection and differential diagnosis of these metabolic diseases have been challenged due to high interindividual variability [116,117,118]. For example, it should be noted that not all individuals afflicted with obesity inevitably experience subsequent metabolic abnormalities; rather, a notable proportion of approximately 25 to 40% can maintain a state of good health over the course of their lifetimes [116,117,119]. The stage-specific BA perturbation patterns may provide a new sight for monitoring the risk of several metabolic diseases such as NAFLD and T2DM. However, to date, it remains to be seen whether these findings can be translated into clinical practice. Meanwhile, a few investigators have challenged this assertion and indicated that there is currently insufficient evidence to support BAs as biomarkers. For example, the researcher followed 205 patients with impaired fasting glucose for 5 years in the IT-DIAB cohort study and found that fasting plasma BAs are not useful clinical biomarkers for predicting the non-obese diabetic in individuals with impaired fasting glucose, but an unexpected association between 6α-OH BAs and glucose parameters was associated with metabolic homeostasis [120]. Indeed, BAs are highly impacted by numerous factors and are stage and/or population specific. More research therefore is needed to reveal stage-specific BA perturbation patterns and provided novel biomarkers and tools for monitoring the progression of metabolic and inflammatory disorders, such as NAFLD, T2DM, and IBD, as well as cancer.

## 7. Conclusions

Overall, the alterations in BA profiles are involved in the development of chronic subclinical inflammation and even metabolic disorders, especially in the earlier stages. Most studies so far have focused on how different BA species as hormones that activate several distinct downstream pathways to modulate glucose and lipid metabolism as well as inflammatory responses. It will be equally important to investigate the gut microbiota interacting with BAs and altering BA metabolism for dissecting “gut–liver axis” communication process. Moreover, extending findings from rodents to humans is tempting. However, species-related differences would be expected between model animals and humans, since the different gut microbiota composition and immune systems [121]. Another consideration is that the composition of BAs differs between rodents and humans. As noted above, β-MCA dominates the BA pool in rodents and functions of antagonizing FXR, whereas CDCA is a major BA in humans which has the opposite effect [19,100]. It follows that, further work is required to check whether these rodent findings are appropriate to humans, and more clinical research still needed to identify the characteristic features of patient BA profiles, which may better permit an accurate clinical diagnosis with ancillary testing.

## Figures and Tables

**Table 1:** The profile of bile acid changes varied in different patients.

Disease	Bile Acid Alterations	Ref.
Non-alcoholic fatty liver disease (NAFLD)	↑Serum GCA:TCA ratio, GDCA:TDCA ratio, GCDCA:TCDCA ratio; ↓Serum non-12α-OH BAs, such as UDCA and THDCA.	[44,45]
Non-alcoholic steatohepatitis (NASH)	↑Serum conjugated 12α-OH-BAs such as TDCA and GDCA; ↑Serum 12α-OH BAs (patients with fibrosis); ↓Serum non-12α-OH BAs (patients without fibrosis).	[46,47,65]
Type 2 diabetes mellitus (T2DM)	↑Serum 12α-OH BAs; ↓Serum non-12α-OH BAs, including HCA species.	[24,49,66,67,68]
Hepatocellular carcinoma (HCC)	↑Serum TBA, mainly in conjugated PBAs including GCA, GCDCA, TCA, TCDCA, and TUDCA (early-stage HCC patients with cirrhosis); ↓Serum 12α-OH BAs:non-12α-OH BAs ratio (patients with advanced HCC).	[51,52,53]
Inflammatory bowel diseases (IBD)	↑Fecal conjugated BA; Conjugated PBAs; 3α-OH-sulfated SBAs; ↓Fecal SBA, especially TGR5-specific BAs; *3-oxo*LCA and *isoallo*LCA.	[16,55,69,70]
Colorectal cancer (CRC)	↑Fecal SBA, mainly in DCA and LCA in early-stage	[54,57,71]
Primary biliary cholangitis (PBC)	↑Serum PBA, such as CA, CDCA, and their conjugates; ↓Serum SBA, such as DCA and its conjugates, LCA and TLCA; ↑Fecal PBA, such as Fecal CA, TCA, CDCA and TCDCA; ↓Fecal LCA and GDCA.	[59,62]
Primary sclerosing cholangitis (PSC)	↑Serum TBA; ↑Serum CA, CDCA and their conjugates, UDCA, TDCA; ↓Serum LCA, TLCA, DCA and GDCA.	[61,62,63]
Intrahepatic cholestasis of pregnancy (ICP)	↑Maternal serum TBA	[64]

## Data Availability

The datasets presented in this study can be found in online repositories. The names of the repository/repositories and accession number(s) can be found in the article.

## Abbreviations

ASBT, apical sodium-dependent bile acid transporter; BA, bile acid; BAAT, bile acid-CoA amino acid N-acyltransferase; BACS, bile acid CoA synthetase; BSEP, bile salt export pump; BSH, bile salt hydrolase; CA, cholic acid; CDCA, chenodeoxycholic acid; CYP450, cytochrome P450 enzymes; CYP7A1, cholesterol 7 alpha-hydroxylase; CYP7B1, oxysterol 7 alpha-hydroxylase; CYP8B1, sterol 12 alpha-hydroxylase; CYP27A1, sterol 27-hydroxylase; DCA, deoxycholic acid; FXR, farnesoid x receptor; GCA, glycol-cholic acid; GCDCA, glycol-chenodeoxycholic acid; HCA, hyocholic acid; HDCA, hyodeoxycholic acid; HFD, high-fat diet; HSDH, hydroxysteroid dehydrogenase; LCA, lithocholic acid; MRP2/3/4, multidrug resistance-associated protein 2/3/4; NTCP, sodium-dependent taurocholate co-transporting polypeptide; OATP1, organic anion transporter 1; OSTα/β, organic solute transporter alpha/beta; TBA, total bile acid; TCA, tauro-cholic acid; TCDCA, tauro-chenodeoxycholic acid; TGR5, Takeda G-protein coupled receptor; UDCA, ursodeoxycholic acid; α-MCA, α-muricholic acid; β-MCA, β-muricholic acid; ω-MCA, ω-muricholic acid.
